# A Retrospective Chart Review of Treatment Seeking Middle Aged Individuals at a Tertiary Care Substance Use Disorder Treatment Centre in North Part of India over Five Successive Years: Findings from Drug Abuse Monitoring System

**DOI:** 10.1155/2013/316372

**Published:** 2013-10-27

**Authors:** Yatan Pal Singh Balhara, Ashwani Mishra, Hem Sethi, Rajat Ray

**Affiliations:** Department of Psychiatry, National Drug Dependence Treatment Centre (NDDTC), All India Institute of Medical Sciences (AIIMS), New Delhi 110029, India

## Abstract

Adolescents and young adults continue to remain the main focus of attention with regards to substance use related problems. There has been a limited focus on illicit substance use among middle aged and elderly population. The current study explored the changing trends of substance use among treatment seeking middle aged individuals (aged 40–60 years) at a tertiary level drug dependence treatment centre. The questionnaire used to gather information for the study is a 19-item structured questionnaire. It includes information on various sociodemographic variables, “current,” and “ever” use of substance. Information is also collected on variables related to high risk injecting drug use and HIV status of the individuals. There has been consistent increase in the population of treatment seekers over five years. Over the five-year period, the absolute percentage increase in treatment seeking population is approximately 21%. Polysubstance use was found to increase significantly over five-study years (*P*_Trend_ = 0.007).

## 1. Introduction

Substance use disorders have been recognised as a major public health problem globally including India. Psychoactive substance use has typically been associated with onset during late adolescence or early adulthood [1]. Majority of substance users in surveys across different countries are adolescents and young adults [2, 3]. Nationwide survey on psychoactive substance use in India, has found around 70% of current users to be aged 40 years or less [4]. Those in middle years of life and elderly population constituted the remainder of 30% of current users. Consequently, adolescents and young adults continue to remain the main focus of attention with regards to substance use related problems [5].

While adolescents and young adults constitute substantial proportion of the current substance users, the consequences of use during these years continue to impact the middle age and later years of life. Various complications related to use of psychoactive substances are likely to impact individuals in middle years of life [6]. There has been a limited focus on illicit substance use among middle aged and elderly population [7, 8].

Deaddiction centres established under the Drug DeAddiction Programme, Ministry of Health and Family Welfare, Government of India, continue to be the key service provider for individuals diagnosed with these disorders in the country [9]. There is limited published literature from India regarding time trends of change in profile of treatment seeking substance using population [10–13]. However, none of these reports focused on middle aged population. The current study explored the changing trends among treatment seeking middle aged individuals (aged 40–60 years) at a tertiary level drug dependence treatment centre in the north part of India.

## 2. Materials and Methods

### 2.1. Setting

The study reports finding from a tertiary level substance use disorders treatment centre in the north part of India.

### 2.2. Study Type

The present study is based on retrospective chart review of data collected through the “Drug Abuse Monitoring System (DAMS)” database. The data were collected for all new consecutive treatment seekers reporting to the centre through a structured questionnaire [9].

### 2.3. Study Questionnaire

The questionnaire used to gather information for the study is a 19-item structured questionnaire. Information was collected using the same questionnaire over the years. It included information on various sociodemographic variables including age, gender, educational qualification, occupational status, and living arrangement. Both “current” (use in the past 30 days) and “ever” use (use ever in life time) of substance are assessed by this questionnaire. Information is also collected on variables related to high risk injecting drug use and HIV status of the individuals.

The information is entered in the appropriate database which is regularly updated. In the present study, the data over a 5-year period (2007–2011) has been analysed and presented. The study group was compared with the rest of the treatment seekers to check for the comparability of reported substances use.

### 2.4. Statistical Analysis

The data were analysed using SPSS version 21.0. The data distributions for various substances of ever and current usage are presented through line diagram. The chi-square test for trend was applied to test for the trend over period of five years. The Normal Z test was applied to test for the significant difference in the proportion of various current and ever use of substances, IDU (current and ever), between the age groups 40–60 years and others for all years. The two-sided *P* < 0.05 was considered statistically significant.

## 3. Results

A total of 3071 treatment seekers in the age group of 40–60 years have been included in the analysis. There has been consistent increase in the population of treatment seekers over five years, except in 2009, which demonstrated a slight fall to 549 from baseline figure of 558 in the year 2007. Over the five-year period, the absolute percentage increase in treatment seeking population is approximately 21% (2007 : 558 versus 2011: 674). Roughly, every three in ten (29.9%) and seventeen in hundred (17.3%) treatment seeker has educational attainment to secondary level and graduation, respectively. At the time of assessment, only a small proportion was unemployed (3.7%). Majority of the treatment seekers were employed. Full-time and part-time employments were observed in 24.4% and 29.8% of the treatment seekers, respectively. Largest proportion of treatment seekers (59.2%) belonged to nuclear family (Table 1).

Slightly less than half of the treatment seekers reported “current” alcohol use (44.3%). In addition to alcohol, “current” use was reported for tobacco (84.3%), heroin (42.5%), cannabinoids (17.3%), and opium (15.8%). “Ever” use of alcohol was reported by 72.0% of the treatment seekers. “Ever” use of heroin and cannabinoids was reported by 45.1% and 28.7% of the treatment seekers, respectively.

No significant change in time trends was observed for any of the psychoactive substances. However, polysubstance use (ever) (defined as use of more than one substance excluding tobacco) was found to increase significantly over the five-study years (2007-22.6%, 2008-28.1%, 2009-22.0%, 2010-26.5%, and 2011-30.9%; *P*_Trend_ = 0.007).

Every one in ten (9.5%) of the treatment seekers was injecting drug users (IDUs), and out of them, about every fifth (21.8%) reported sharing of needles and/or syringes. Sharing of paraphernalia was also reported by one-third of IDUs (30.4%). Most of the IDU preferred IV route (75.1%). The rest injected through IM route (Table 2).

Roughly, one in fifty (1.8%) reported to have underwent HIV screening. Of these who had been screened, 10.9% reported to be seropositive for HIV. 

**Figure 1:**
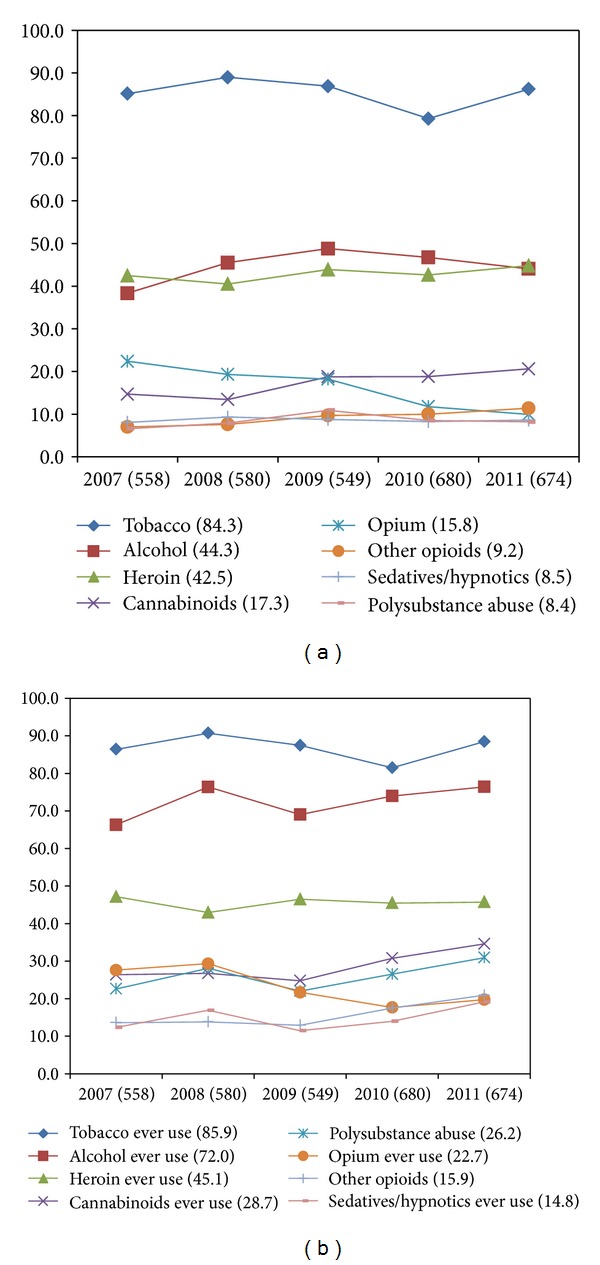
(a) Line diagram showing percentage distribution of common substances of abuse (current) in the respective years. Figures after the parentheses on *x*-axis denote total treatment seeking population in that year and the year after in legend showing the total percentage. (b) Line diagram showing percentage distribution of common substances of abuse (ever) in the respective years. Figures after the parentheses on *x*-axis denote total treatment seeking population in that year and the year after in legend showing the total percentage.

The distributions of common substances reported (current and ever use) over the successive years are depicted in Figures 1(a), and 1(b).

The study group was compared with the rest of the treatment seekers to check for the comparability of reported substances use. The analysis was performed using cross tabulation of “current” and “ever” use for all substances and IDU. The 40–60 years age group subjects, as compared to other age group, had significantly (*P* < 0.05) higher “current” use of alcohol (for years 2008, 2010, and 2011) and opium for all five-study years. However, this age group had significantly (*P* < 0.05) lesser “current” use for other opioids, cannabinoids, and volatile solvents for all years. Tobacco “current” use for 40–60 years age group was significantly less for the year 2009 only.

The same directionality in the respective years as mentioned above was also noted for “ever” use of alcohol, opium, other opioids, cannabinoids, and tobacco. Additionally, “ever” use of sedatives was significantly lower in 40–60 years age group for the year 2007. This age group had significantly lesser “current” and “ever” IDU for all five-study years.

## 4. Discussion

The current study explored the time trends among treatment seeking middle aged individuals at a tertiary level drug dependence treatment centre from the north part of India.

There is limited literature focusing on middle aged individuals for substance use related problems. The World Drug Report (an annual publication of UNODC) [2] and Global Status Report on Alcohol and Health (a publication of WHO) [3] provide the overall trends across all age groups (aged 15 years and more). Additionally, these reports focus specifically on information on prevalence and trends among youth. However, middle aged population is not covered separately in these reports.

There are few published reports on, change in, profile of treatment seekers for substance use disorders across different years from India [10–13]. However, none of these reports have analysed data over successive years. Also they have not analysed the data for specific age groups. Consequently, there is no published literature on time trends among treatment seeking middle aged individuals from the country.

The world literature has consistently documented a comparative lower prevalence of substance use disorders among middle aged individuals in general population based studies. The Epidemiologic Catchment Area (ECA) study in USA reported that 7% of persons in 45–64 years age group had a lifetime prevalence of illegal drug use. Active use of illegal drugs occurred in 0.8% of subjects aged 45–64 years [14]. In the first National Survey in India 30% of all current users of psychoactive substance were 40 years or more [4].

However, it is important to study substance use related disorders among the middle aged population for various reasons. First, while middle aged individuals constitute a smaller fraction of all substance users in general population based surveys, the same is not true for studies conducted across different settings. For example, in a study of substance abuse by offenders in USA prison, seventy-one percent of middle aged inmates reported substance abuse problems, which was higher than the mean value [15]. Second, the pattern and type of substance use tend to vary across different age groups. Older adults are more likely to use alcohol and less likely to be injection drug users and heroin, cocaine, and polysubstance users as compared to younger adults [16]. Third, middle aged substance users tend to have a different sociodemographic profile as compared to the young users. Older adults tend to come from a more stable environment in terms of income and marriage stability [17]. Fourth, these individuals are less likely to be referred by healthcare workers [18]. Fifth, detection of substance abuse problem tends to differ among the middle aged individuals as compared to young aged individuals as it is often identified initially when patients present with medical problems secondary to substance use [17]. Sixth, with the increase in life expectancy, these individuals are likely to constitute a larger fraction of treatment seekers in the coming years.

Increase in availability of treatment services for young substance users coupled with chronic relapsing nature of the problem is likely to add to the cohort of middle aged substance users in many countries. An increase in mean age of IDUs has been reported in some studies. An analysis of data from 1979 to 2002 from USA, found that the mean age of participants with IDU within the past year increased from 21 to 36 years. Also the mean age of participants with IDU ever increased from 26 to 42 years. From 2000 to 2002, 59.4% of all persons with IDU ever were 35 to 49 years in this study [19].

An overwhelming majority of subjects aged 40–60 years across all years in the current study were found to be married (minimum of 76.3% in year 2010). This is an interesting finding. Substance use disorders have been associated with significant marital discord and high rates of separation and divorce in western settings [20]. Western studies have also found older substance using adults in treatment services to come from a more stable environment in terms of marriage stability [17]. The findings of the current study reflect possibility of a relatively less impact of substance use disorder on marital status. However, the observation could simply be a result of more likelihood of those with good marital support to seek treatment. However, the proportion of married individuals in the current study is higher than that reported by a previous study from another deaddiction centre in India. The proportion of married individuals varied from 62.5% to 76.8% across different years in this study [12, 20].

Similarly, majority of the treatment seekers (~80%) were literate in the current study. This observation could also be a reflection of greater help seeking from this treatment set-up by literate individuals. However, literature from west suggests that years of education, marital status, and employment status are unlikely to influence treatment seeking by substance users [21–23].

Around one-fourth of the subjects in the current study were found to be unemployed. Western studies have found that users of “hard” drugs such as heroin and crack cocaine are significantly less likely to be employed than other adults of working age [24].

The trends of “current” as well as “ever” use for most of the psychoactive substances remained stable across the five-study years. The notable exceptions to this observation were current opium use, current as well as ever use of other opioids, and current as well as ever cannabis use. Interestingly, a previous Indian study reported a reduction in proportion of treatment seekers with alcohol use (from 57.3% to 47.8%) and a significant increase in those reporting opioid use (from 36.8% to 53.2%) across three decades. The percentage of respondents with opioids as primary drug of use increased from 9.5% (in 1980) to 73.61% (in 2002), and percentage of respondents with alcohol as primary drug of use increased from 4.8% (in 1980) to 9.72% (in 2002) [12]. Another study from the northern part of the country reported an increase in percentage of subjects presenting with alcohol and opioid use [11]. Sachdev et al. [13] reported a decline in percentage of alcohol users from 32.80% (in 1994) to 14.57% (in 1998). Venkatesan and Suresh [10] also reported a small decline in alcohol users from 87.2% (in 1985) to 79.6% (in 2005).

The finding of reduction in users of opium was also observed in the study by Basu et al. [12]. This study reported a significant decline in opium users across the study period of three decades (from 47.4% to 18.3%). Sachdev et al. [13] also reported a decline in percentage of opium users from 14.71% (in 1994) to 8.52% (in 1998). However, this study reported an increase in users of poppy husk from 13.50% (in 1994) to 34.36% (in 1998).

The findings of this study differed from previous Indian studies with regards to cannabis use. Basu et al. [12] reported a decline in cannabis use across three decades (from 13.5% to 9.6%) as opposed to an increase in the current study (from 14.7% to 20.6%). Similarly, Margoob et al. [11] reported a reduction in percentage of respondents citing cannabis as primary drug of abuse (from 77.8% in 1988 to 16.66% in 2002). Findings of high proportion of tobacco users and low proportion of inhalant and stimulant users were common across the current study and the study by Basu et al. [12].

Polysubstance use was found to increase significantly over the five-study years. Polysubstance use (use of two or more substances) was also found to increase significantly in earlier studies from India [10–12]. Basu et al. [12] reported an increase in polysubstance use from 8.7% to 62.7% over three decades. Margoob et al. [11] reported an increase from 15.8% to 41.66% over a period of two decades. Venkatesan and Suresh [10] also reported a significant increase in polysubstance users from 12.8% (in 1985) to 20.4% (in 2005). However, Sachdev et al. [13] reported a decline in percentage of polysubstance users from 4.62% (in 1994) to 2.23% (in 1998).

In spite of a decline in use of opium, the overall opioid use remained stable across the five years in the current study. This reflects an increase in use of other opioids. The group of “other opioids” comprises of prescription opioids such as dextropropoxyphene and codeine and injection opioids such as pentazocine and buprenorphine.

In a secondary analysis of the National Surveys on Drug Use and Health (NHSDU) data in USA for the year 2005-2006, it was reported that around 65% of subjects used alcohol, 3.89% marijuana, 0.08% heroin, and 0.13% inhalants during the past year among those aged 50–64 years [14]. Although the age of subjects in these two studies differed slightly, heroin, opioid, and cannabis use were higher in the current study.

Prevalence of alcohol and opium use was higher and that of other opioids and cannabis was lower among individuals aged 40–60 years as compared to other age groups in the current study. Western studies have also found that older adults are more likely to use alcohol and less likely to be injection drug users and heroin, cocaine, and polysubstance users as compared to younger adults [16].

Individual in 40–60 years age group in the current study had significantly higher “current” use of opium. Higher use of opium in this age group as compared to other age groups seems to be a reflection of changing epidemiology of opioid use in the country. It has been argued that excessive control of opium use had led to increased use of illicit opioids including heroin in South East Asia [25]. Recreational as well as habitual use of opium has been reported from various South Asian countries including Afghanistan [26], China [27, 28], Iran [29], India [4], Myanmar, and Lao [30]. In addition to these countries, opium use has also been reported from Thailand, Bangladesh, Nepal, and Sri Lanka [25]. Stricter control of opium, increased availability of heroin, and increased use of other opioids in the country over the years are also reflected in treatment seeking substance users. A significantly higher use of opium was observed in older age group (40–60 years) in the current study. Additionally, a gradual decline in opium users within this age over the five-study years was also noted. An additional finding of concern is the gradual increase in users of “other opioids” over the years in this age group as most of these “other opioids” are abused through injecting route as well. It is likely that opioid users are shifting from opium to potentially more harmful opioids in the country.

No specific patterns were observed for IDU related variables in this study. Overall, 22% of the IDUs reported sharing of needles. Majority of the respondents (75%) were injected through the intravenous route; the rest were injected intramuscularly. There was a decline in percentage of individuals reporting sharing (from 39.1% in 2007, to 17.1% in 2011). Only 1.8% of these individuals reported getting tested for HIV. Of these, 10% reported to be seropositivity for HIV infection. HIV/AIDS continues to be a concentrated epidemic in the country with a seropositivity rate of 9.19% among IDUs [31]. States of Punjab and Delhi (constituting part of the catchment area for this treatment centre) have reported a higher HIV prevalence among IDUs as compared to the national average. The findings of significantly lower “ever” and “current” IDU among 40–60 years age group as compared to other age groups are also a possible reflection of the cohort effect. IDU (particularly of opioids) is believed to be stared later as compared to use of opioids through other routes such as inhalation and chasing in the country.

The current study presents the time trends (over a five-year period) of the profile of treatment seeking middle aged psychoactive substance users at a treatment centre in north part of India. Reported use of alcohol, tobacco, inhalants, and sedatives has remained stable over these years. Reported use of cannabis has increased over the years. Cannabis use has been associated with physical as well as psychological complications [32]. While use of opium has declined over these five years in the current study, an increase in use of “other” opioids which include pharmaceutical opioids (including injection formulations) presents a challenge for future. Another challenge is to cater to the needs of a high proportion of IDUs among the opioid users.

The current study is based on secondary analysis of data. Use of such methodology is associated with certain limitations. While the time trends have been reported, the current study did not explore the possible reasons for these trends. Also while the findings reflect the profile of treatment seeking individuals, it does not necessarily reflect prevalence of use of these psychoactive substances in general population. However, when interpreted in light of the general population estimates of psychoactive substance use, the findings could help to plan services for these individuals.

## Figures and Tables

**Table 1:** Sociodemographic variables of the treatment seeking middle aged individuals over the 5-year period.

Variable	2007 (558)		2008 (580)		2009 (549)		2010 (680)		2011 (674)		Total	
	*n*	%	*N*	%	*n*	%	*n*	%	*n*	%	*n*	%
Marital status												
Never married	51	9.1	42	7.2	44	8	63	9.3	51	7.6	251	8.2
Married	473	84.8	495	85.3	491	89.4	519	76.3	550	81.6	2528	82.3
Divorce/separated	5	0.9	7	1.2	1	0.2	17	2.5	15	2.2	45	1.5
Widow/widower	17	3	26	4.5	8	1.5	21	3.1	25	3.7	97	3.2
Separated due to drug abuse	10	1.8	10	1.7	5	0.9	5	0.7	21	3.1	51	1.7
Not known	2	0.4	0	0	0	0	55	8.1	12	1.8	69	2.2
Educational qualification												
Illiterate	132	23.7	125	21.6	101	18.4	129	19	170	25.2	657	21.4
Literate	41	7.3	42	7.2	58	10.6	41	6	37	5.5	219	7.1
Primary	95	17	64	11	77	14	59	8.7	78	11.6	373	12.1
Middle	88	15.8	106	18.3	155	28.2	284	41.8	285	42.3	918	29.9
Up to 10th/12th	138	24.7	154	26.6	86	15.7	61	9	91	13.5	530	17.3
Graduation	45	8.1	57	9.8	19	3.5	0	0	0	0	121	3.9
PG/tech/prof.	17	3	32	5.5	26	4.7	23	3.4	13	1.9	111	3.6
Not known	2	0.4	0	0	27	4.9	83	12.2	0	0	112	3.6
Employment status												
Never employed	16	2.9	6	1	16	2.9	12	1.8	9	1.3	59	1.9
Presently unemployed	171	30.6	128	22.1	120	21.9	153	22.5	167	24.8	739	24.1
Full-time employed	160	28.7	141	24.3	139	25.3	163	24	145	21.5	748	24.4
Part-time employed	39	7	110	19	189	34.4	282	41.5	288	42.7	908	29.6
Self-employed	159	28.5	186	32.1	73	13.3	2	0.3	49	7.3	469	15.3
Student	0	0	1	0.2	0	0	0	0	0	0	1	0.0
House wife/girl	6	1.1	4	0.7	3	0.5	6	0.9	6	0.9	25	0.8
Any other	4	0.7	4	0.7	7	1.3	5	0.7	10	1.5	30	1.0
Not known	3	0.5	0	0	2	0.4	57	8.4	0	0	62	2.0
Living arrangement												
Joint family	94	16.8	201	34.7	212	38.6	164	24.1	207	30.7	878	28.6
Nuclear family	433	77.6	350	60.3	286	52.1	386	56.8	364	54	1819	59.2
Alone	17	3	24	4.1	25	4.6	40	5.9	47	7	153	5.0
With friends	3	0.5	3	0.5	4	0.7	4	0.6	4	0.6	18	0.6
Any other	6	1.1	2	0.3	7	1.3	2	0.3	3	0.4	20	0.7
Not known	5	0.9	0	0	15	2.7	84	12.4	49	7.3	153	5.0

**Table 2:** IDU related variables of the treatment seeking middle aged individuals over the 5-year period.

Variable	2007 (558)		2008 (580)		2009 (549)		2010 (680)		2011 (674)		Total	
	*n*	%	*N*	%	*n*	%	*n*	%	*n*	%	*n*	%
IDU ever	81	14.5	71	12.2	85	15.5	99	14.6	106	15.7	442	14.4
IDU current	46	8.2	40	6.9	55	10.0	82	12.1	70	10.4	293	9.5
Sharing of needles	18	39.1	15	37.5	11	20.0	8	9.8	12	17.1	64	21.8
Sharing of needles by IV route	19	41.3	34	85.0	51	92.7	59	72.0	57	81.4	220	75.1
Sharing of needles by IM route	26	56.5	6	15.0	4	7.3	18	22.0	13	18.6	67	22.9
Sharing of needles (Route not known)	1	2.2	0	0.0	0	0.0	5	6.1	0	0.0	6	2.0
Paraphernalia	5	10.9	34	85.0	42	76.4	6	7.3	2	2.9	89	30.4
HIV screening	10	1.8	4	0.7	5	0.9	5	0.7	31	4.6	55	1.8
